# Neck circumference as a predictor of all-cause mortality in middle-aged and older adults in rural Ecuador

**DOI:** 10.1093/inthealth/ihad119

**Published:** 2024-01-17

**Authors:** Oscar H Del Brutto, Denisse A Rumbea, Maitri Patel, Robertino M Mera

**Affiliations:** School of Medi cine and Research Center, Universidad Espíritu Santo – Ecuador. Via Puntilla-Samborondon Km 2.5, Samborondón 09-01-952, Ecuador; School of Medi cine and Research Center, Universidad Espíritu Santo – Ecuador. Via Puntilla-Samborondon Km 2.5, Samborondón 09-01-952, Ecuador; School of Medicine, University of Virginia, Charlottesville, VA 22903, USA; Biostatistics/Epidemiology, Freenome, Inc., South San Francisco, CA, USA

**Keywords:** mortality, neck circumference, population-based longitudinal study, rural settings

## Abstract

**Background:**

Neck circumference (NC) has been associated with mortality secondary to cardiovascular diseases and other conditions. However, information on this association in the population at large is limited. We aimed to assess this association in community dwellers living in rural Ecuador.

**Methods:**

Individuals aged ≥40 y who were enrolled in the population-based Three Villages Study cohort were prospectively followed to estimate mortality risk according to baseline measurements of NC, after adjusting for relevant confounders.

**Results:**

Analysis included 1521 individuals followed for a mean of 6.4±3.4 y. Mean NC was 36.2±3.7 cm, with 509 (33%) individuals allocated to the first (25–34 cm), 319 (21%) to the second (36–37 cm), 417 (27%) to the third (37–39 cm) and 276 (18%) to the fourth (40–50 cm) quartile. A total of 211 (14%) individuals died during the follow-up. Overall, the crude mortality rate was 2.3 per 100 person-years, which increased to 5.63 for those in the fourth NC quartile. An adjusted Cox-proportional hazards model showed that individuals in the fourth quartile of NC had higher mortality risk compared with the first quartile (HR: 2.98; 95% CI 1.77 to 5.02).

**Conclusion:**

Larger NC increases mortality risk in middle-aged and older adults of indigenous ancestry living in rural Ecuador.

## Introduction

Increased neck circumference (NC) is an indicator of augmented fat in the upper body and has been signaled as a risk factor for several non-communicable diseases.^1,2^ This anthropometric measurement has been used as a predictive marker of incident stroke, coronary artery disease, atrial fibrillation, diabetes mellitus and obstructive sleep apnea, among other conditions,^3–7^ but these results have not been replicated in other studies.^8^ A link between increased NC and poor outcomes in certain diseases such as intracranial hemorrhages has also been demonstrated.^9^ In addition, previous cross-sectional studies conducted in representative samples of the population recruited for the current study showed significant associations between NC and obstructive sleep apnea and the carotid intima-media thickness.^10,11^

To the best of our knowledge, no study has addressed the association between NC and mortality in Ecuador or in most of Latin American countries, with the possible exception of a Brazilian study showing increased mortality after 1 y of follow-up among individuals with a stroke who also have increased NC.^12^

NC has been reported to have an effect on mortality risk associated with some of the above-mentioned disorders. However, information on the independent importance of an increased NC as a predictor of mortality in the population at large is limited.^8,12,13^ Information from the literature suggests that NC is not directly associated with mortality but exerts an effect on patients with certain conditions or diseases.

Using the population-based Three Villages Study cohort, this longitudinal prospective study aims to assess the relationship between NC and all-cause mortality in community-dwelling middle-aged and older adults of indigenous ancestry living in rural Ecuador.

## Materials and Methods

### Study population

This study was conducted in community dwellers aged ≥40 y living in Atahualpa, El Tambo and Prosperidad, which are three neighboring villages located in rural areas of coastal Ecuador. Overall, the population is homogeneous regarding ethnicity (Amerindian ancestry), low levels of education, lifestyles, dietary habits and comparable cardiovascular risk factors, as detailed in prior studies.^14^ In addition, the migration rate is low and adherence to the study is high, which provides an optimal setting for conducting cohort studies.^15^

### Study design

Following a longitudinal prospective design, study participants were identified by means of annual door-to-door surveys (2012 to 2019) and enrolled in the Three Villages Study cohort. All individuals signed a comprehensive informed consent form at the time of enrollment. NC, demographics and cardiovascular risk factors were determined at baseline. Field personnel visited study participants every 3 mo to determine permanence into the study and to monitor their vital status. Those who emigrated or declined consent were removed from the cohort at the administrative censoring date of the last annual survey when the individual was interviewed. Individuals who died were censored on the day of death, which was confirmed by reviewing death certificates. The last administrative censoring date was set as 1 June 2023. The study was approved by the Ethics Committee of Hospital-Clínica Kennedy, Guayaquil (FWA 00030727). Aggregated data from this study will be available from the corresponding author upon reasonable request.

### Neck circumference

With the individual seated, the NC (in centimeters) was measured with the use of a stretch-resistant tape placed immediately above the cricoid cartilage and perpendicular to the long axis of the neck. All measurements were taken twice and an average of each of them was used for analyses. To reduce the possibility of misclassifications due to poorly defined cut-offs for NC in individuals of different ethnic groups, we used continuous values of NC (further stratified in quartiles) for analyses.

### Covariables investigated

Demographics, level of education and cardiovascular risk factors were assessed at baseline and designated as potential confounders. Demographics (age, sex) and level of education (up to primary school education or higher) were recorded by self-report. Cardiovascular risk factors were assessed according to the Life's Simple 7 construct of the American Heart Association,^16^ which has been widely used in people living in both urban and rural settings and has demonstrated to be reliable regardless of differences across regional disparities in cardiovascular risk factors.^17–19^ The Life's Simple 7 is composed of seven health metrics, which are categorized in the poor range according to well-defined parameters, as follows: (1) current smoker or had stopped for <1 y; (2) body mass index of ≥30 kg/m^2^; (3) no moderate or vigorous physical activity; (4) ≤1 component of the five healthy diet proposed by the American Heart Association (≥4.5 cups of fruits and vegetables/day, ≥two 3.5-oz servings of fish/week, ≥three 1-oz equivalent servings fiber-rich whole grains/day, <1500 mg sodium/day and ≤450 kcal sugar-sweetened beverages/week); (5) blood pressure ≥140/90 mmHg; (6) fasting glucose ≥126 mg/dL; and (7) total cholesterol blood levels ≥240 mg/dL.^16^

### Statistical analysis

All analyses were carried out using STATA version 17 (College Station, TX, USA). In unadjusted analyses, continuous variables were compared by linear models and categorical variables by the χ^2^ or Fisher exact test. To calculate the person-years of follow-up we considered the time from enrollment to the last administrative censoring date, study drop-out or the day of death. Poisson regression models were fitted to estimate the overall crude mortality rate and comparisons for mortality rate according to NC stratified in quartiles. Cox-proportional hazards models, adjusted for demographics, level of education and cardiovascular risk factors, were fitted to calculate the HR with its 95% CI, to estimate the risk for all-cause mortality according to quartiles of NC. Linear splines for continuous NC values were used to estimate the discontinuity of the slopes for the mortality rate.

## Results

Of 1543 Atahualpa, El Tambo and Prosperidad residents aged ≥40 y enrolled in the Three Villages Study cohort from June 2012 to June 2019, 1521 (99%) received baseline interviews and procedures for assessment of the investigated variables and were included in this cohort. The remaining 22 individuals either declined consent to participate (n=12) or had missing baseline clinical forms (n=10). There were no differences in clinical characteristics across individuals who participated in this study and those who did not. The total follow-up in the population was 9699 person-years and the mean follow-up was 6.4±3.4 y. Individuals censored before the end of the study were those who emigrated (n=54), declined further consent (n=78) or died (n=211); however, they also counted towards the total time of follow-up.

For a total of 9699 person-years of follow-up, we needed at least 196 deaths to obtain an HR of 1.66 at the alpha level of 0.05 and with 80% power. Therefore, our sample had sufficient observed outcomes to detect differences in the relationship between NC and all-cause mortality.

The mean (±SD) age of study participants at enrollment was 56.5±12.5 (median: 56) y, 850 (56%) were women and 943 (62%) had primary school education only. Cardiovascular metrics in the poor range were as follows: smoking status: 74 (5%); body mass index: 444 (29%); physical activity: 148 (10%); diet: 163 (11%); blood pressure: 474 (31%); fasting glucose: 374 (25%); and total cholesterol blood levels: 160 (11%). The mean (±SD) NC was 36.2±3.7 (median: 36) cm, with 509 individuals allocated to the first quartile (25–34 cm), 319 to the second quartile (35–36 cm), 417 to the third quartile (37–39 cm) and the remaining 276 to the fourth quartile (40–50 cm).

In unadjusted analysis, individuals who had a larger NC were younger at baseline and less often women than those allocated to lower NC quartiles. Several cardiovascular metrics in the poor range (smoking status, body mass index, diet and blood pressure) were more frequent among subjects with a larger NC, while high total cholesterol blood levels were less frequent among subjects allocated to the fourth quartile of NC (Table 1).

**Table 1.** Clinical characteristics of 1521 community dwellers aged ≥40 y enrolled in this study according to quartiles of neck circumference (unadjusted analyses)

	NC 25–34 cm	NC 35–36 cm	NC 37–39 cm	NC 40–50 cm	
	(n=509)	(n=319)	(n=417)	(n=276)	p value
Age (y) at baseline, mean±SD	57.3±13.2	57.6±12.5	56±11.8	54.7±12.1	0.012*
Women, n (%)	450 (88)	210 (66)	142 (34)	48 (17)	<0.001*
Primary school education, n (%)	328 (64)	209 (66)	250 (60)	156 (57)	0.065
Current smoker, n (%)	6 (1)	10 (3)	24 (6)	34 (12)	<0.001*
Body mass index ≥30 kg/m^2^, n (%)	53 (10)	99 (31)	120 (29)	172 (63)	<0.001*
Poor physical activity, n (%)	46 (9)	40 (13)	31 (7)	31 (11)	0.095
Poor diet, n (%)	39 (8)	36 (11)	45 (11)	43 (16)	0.008*
Blood pressure ≥140/90 mmHg, n (%)	135 (27)	100 (31)	144 (35)	95 (34)	0.003*
Fasting glucose ≥126 mg/dL, n (%)	125 (25)	77 (24)	99 (24)	73 (26)	0.870
Total cholesterol ≥240 mg/dL, n (%)	70 (14)	34 (11)	45 (11)	11 (4)	<0.001*

NC, neck circumference.

*Statistically significant result.

**Figure 1.**
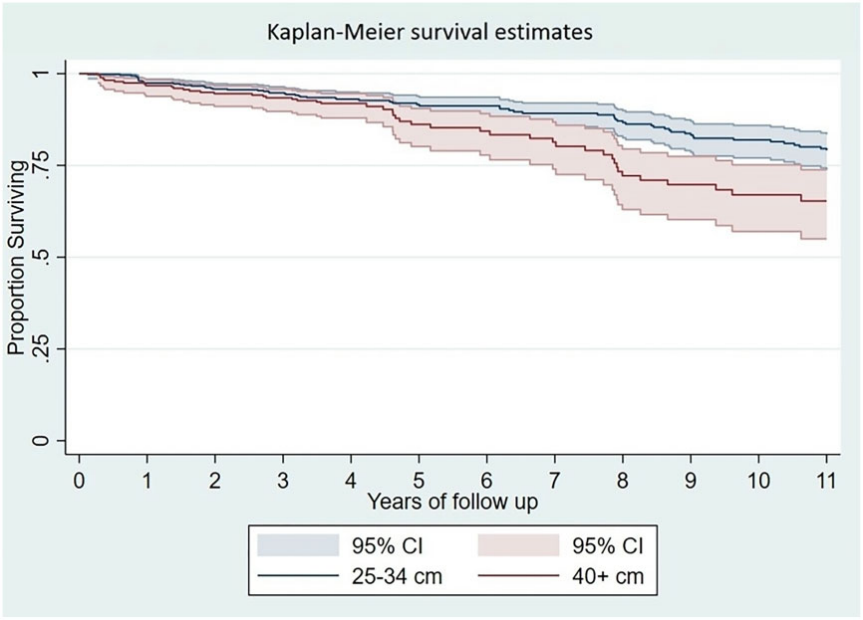
Kaplan–Meier survival curves and HRs with 95% CIs for all-cause mortality according to quartiles of neck circumference. There is a significant difference in mortality across individuals allocated to the fourth quartile compared with those in the first quartile.

A total of 211 (14%) study participants died during the follow-up, resulting in an overall unadjusted crude mortality rate of 2.3 (95% CI 2.2 to 2.7) per 100 person-years. In univariate analysis, a Cox proportional hazards model that considered person-years of follow-up showed a significant increase in mortality risk (HR: 1.66; 95% CI 1.14 to 2.41) among individuals allocated to the fourth quartile of NC compared with those in the first quartile (Table 2). Kaplan–Meier survival estimates showed a significant increased mortality (no overlapping CIs) among individuals in the fourth quartile of NC (Figure 1).

**Table 2.** Unadjusted Cox-proportional hazards model (Breslow method for ties) showing a significantly increased mortality rate among individuals allocated to the fourth quartile of neck circumference compared with those in lower quartiles

Neck circumference	HR	95% CI	p value
Quartile 1 (25–34 cm)	Referent category		
Quartile 2 (35–36 cm)	1.07	0.73–1.56	0.729
Quartile 3 (37–39 cm)	1.00	0.69–1.43	0.990
Quartile 4 (40–50 cm)	1.66	1.14–2.41	0.008*

*Statistically significant result.

In addition, a multivariate Cox-proportional hazards model (adjusted for demographics, level of education and cardiovascular risk factors) confirmed that individuals allocated to the fourth quartile of NC had a higher mortality risk than those in the first quartile (HR: 2.98; 95% CI 1.77 to 5.02). In this model, increased age at baseline, poor physical activity and poor fasting glucose were directly associated with mortality, while a body mass index of ≥30 kg/m^2^ showed an inverse association with the outcome (Table 3).

**Table 3.** Multivariate Cox-proportional hazards model (Breslow method for ties) showing a significantly increased mortality rate among individuals allocated to the fourth quartile of neck circumference compared with those in lower quartiles. In this model, increased age at baseline, poor physical activity and high fasting glucose were directly associated with mortality, while a high body mass index was inversely associated

Neck circumference	HR	95% CI	p value
Quartile 1 (25–34 cm)	Referent category		
Quartile 2 (35–36 cm)	1.24	0.83–1.86	0.297
Quartile 3 (37–39 cm)	1.39	0.89–2.16	0.891
Quartile 4 (40–50 cm)	2.98	1.77–5.02	<0.001*
Age at baseline	1.06	1.04–1.07	<0.001*
Being female	1.20	0.83–1.74	0.325
Primary school education	1.17	0.81–1.70	0.409
Current smoker	0.69	0.28–1.70	0.420
Body mass index ≥30 kg/m^2^	0.58	0.39–0.84	0.005*
Poor physical activity	2.34	1.63–3.35	<0.001*
Poor diet	0.96	0.56–1.65	0.895
Blood pressure ≥140/90 mmHg	1.13	0.85–1.50	0.395
Fasting glucose ≥126 mg/dL	1.92	1.45–2.54	<0.001*
Total cholesterol ≥240 mg/dL	0.59	0.35–1.01	0.055

*Statistically significant result.

**Figure 2.**
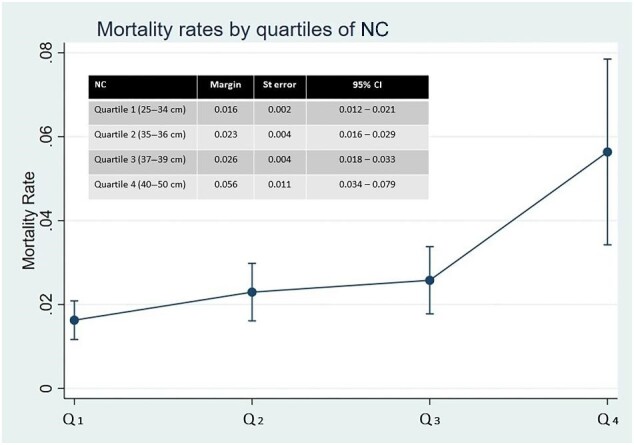
Graph plot showing estimated mortality rates to be significantly higher (no overlapping 95% CIs) in individuals belonging to the fourth quartile of NC compared with those in the lower quartiles.

A fully adjusted Poisson regression model showed estimated mortality rates to be significantly higher in individuals belonging to the fourth quartile of NC compared with those in the lower quartiles (Figure 2). This model showed a non-linear relationship between NC and mortality, with two distinct slopes (linear splines), one that went from 25 to 39 cm and the other from 39 to 50 cm. Replacing categorized NC into linear splines into the same Poisson adjusted model, the mortality rate increased by 6.7% for every cm of increase in NC for individuals in the first linear piece (first, second and third quartiles), and by 22.7% for every cm of increase in NC for those in the second linear piece (fourth quartile).

Using the categorized Poisson regression model, the predicted mortality rate for individuals in the first quartile of NC was 1.63 (95% CI 1.17 to 2.09) per 100 person-years of follow-up, which increased to 5.63 (95% CI 3.42 to 7.85) for those in the fourth quartile. In view of reported differences in the impact of NC on poor outcomes across men and women, we fitted two separate Cox-proportional hazards models (adjusted for relevant covariates), one for men and the other for women, and found no differences in the association between quartiles of NC and mortality across those models (Supplementary file 1).

## Discussion

In this population-based cohort of community-dwelling middle-aged and older adults living in rural Ecuador, a large NC phenotype appears as a significant predictor of all-cause mortality independently of demographics, level of education and traditional cardiovascular risk factors. Interestingly, the association between NC and mortality is not linear, showing a marked increased risk among subjects with a NC of ≥40 cm.

Our results are in line with other studies that have demonstrated an association between increased NC and higher mortality risk related to certain conditions, although studies supporting the independent role of NC on mortality in the general population have resulted in inconsistent findings.^8,12,13^ In a large population cohort of African Americans living in Jackson (Missouri, USA), NC was associated with increased mortality only in adjusted analysis, but the significance diminished after adjustment for demographics and traditional cardiovascular risk factors.^8^ By contrast, the Sleep Heart Health Study—a large longitudinal cohort conducted in community-dwelling adults followed for >10 y—showed NC to be significantly associated with increased mortality among apparently healthy individuals, after adjustment for relevant confounders.^13^ In addition, a small prospective study from Brazil showed that 1-y mortality after a stroke was associated with increasing age and larger NC (>43 cm in men and >38 cm in women),^12^ but 1 y of follow-up was not long enough to reach valid conclusions. With this possible exception, no previous study has addressed the association between NC and mortality in an indigenous Latin American population.

Pathogenesis of the association between NC and mortality is not totally understood; however, recent studies have provided some insights into this relationship. The NC is an important indicator of fat accumulation in the upper body, is more reliable and easier to measure than the waist and hip circumferences, and associates better with cardiometabolic risk factors than other anthropometric indices.^20^ Because of a paracrine effect of adipose tissue and other pathophysiologic mechanisms, fat deposit in the upper body has been shown to correlate with hypertriglyceridemia, increased insulin resistance, high blood pressure, increased biomarkers of inflammation, subclinical atherosclerosis and non-alcoholic fatty liver disease, among other conditions.^1,21–24^ The detrimental effects of abnormal fat deposit in the upper body seem to be independent of other anthropometric indices,^25^ and suggest a rationale behind the correlation between a large NC phenotype and increased mortality risk. These considerations underscore the importance of upper-body fat deposition, and hence the NC as a useful biomarker of cardiovascular risk and subsequent mortality.

The waist circumference, as a determinant of central obesity, has also been shown to be a relevant anthropometric measurement associated with fatal outcomes in some ethnic groups.^26,27^ Therefore, in a post-study analysis we fitted unadjusted and multivariate Poisson regression models to assess this association and found no association between the waist circumference and mortality in the study population (Supplementary file 2).

Our study has limitations. Precise ascertainment of the actual cause of death was not possible in several cases because of suspected inaccuracies in death certificates. It is also possible that some unmeasured confounders may have accounted for at least part of the findings. The study population is confined to individuals of indigenous Ecuadorian ancestry, and the results of the current study may not be generalizable to other ethnic groups. Less than 15% of the population with vascular risk factors received proper treatment during the study years, despite our encouragement on medication compliance. This precluded assessment of the influence of medications on the relationship between the main variables investigated. These limitations are counterbalanced by several strengths of the study, including its population-based longitudinal prospective design, the homogeneity of the study population regarding ethnic background and lifestyle, and the systematic assessment of cardiovascular risk factors by means of standardized protocols that have previously been used and validated in the same population.^14,28^

In conclusion, a NC of ≥40 cm is significantly associated with all-cause mortality in community dwellers aged ≥40 y living in rural Ecuador. Based on our experience comparing characteristics of this study population with other cohorts,^29^ it is possible that the findings of this study may be generalizable to individuals living in different settings. NC measurement is a reliable and practical procedure to identify individuals at risk and to predict deadly outcomes in people living in remote settings with limited access to healthcare. Measures aimed at controlling risk factors associated with increased NC may be challenging in the context of poverty and illiteracy, but may prove cost-effective at reducing mortality risk in underserved populations. Further longitudinal studies are needed to confirm our findings.

## Supplementary Material

ihad119_Supplemental_Files

## Data Availability

Aggregated data will be available upon reasonable request to the corresponding author.
