# Supplementary material for: Neck circumference as a predictor of all-cause mortality in middle-aged and older adults in rural Ecuador
Source: Int Health. 2024 Jan 17;16(6):664–9. doi: 10.1093/inthealth/ihad119 (PMC11532669; doi:10.1093/inthealth/ihad119)
Supplement: ihad119_Supplemental_Files [file ihad119_supplemental_files.zip › Supplementary file 1.docx]

**Supplementary file 1.** STATA outputs of two different Cox proportional hazards models, showing no differences in the association between neck circumference – stratified in quartiles – and mortality across males and females.

**Cox regression – males (observations: 671; deaths: 101)**

| **Neck circumference** | **Hazard ratio** | **95% confidence interval** | ***p* value** |
| --- | --- | --- | --- |
| **Quartile 1 (25 – 34 cm)** | Referent category | | |
| **Quartile 2 (35 – 36 cm)** | 1.91 | 0.76 – 4.79 | 0.169 |
| **Quartile 3 (37 – 39 cm)** | 1.60 | 0.65 – 3.97 | 0.307 |
| **Quartile 4 (40 – 50 cm)** | 4.24 | 1.58 – 11.4 | 0.004* |
| **Age at baseline** | 1.06 | 1.03 – 1.08 | <0.001* |
| **Primary school education** | 0.86 | 0.51 – 1.43 | 0.554 |
| **Body mass index ≥30 kg/m^2^** | 0.91 | 0.83 – 0.99 | 0.023* |
| **Poor physical activity** | 2.15 | 1.24 – 3.73 | 0.006* |
| **Blood pressure ≥140/90 mmHg** | 1.37 | 0.91 – 2.07 | 0.132 |
| **Fasting glucose ≥126 mg/dL** | 2.22 | 1.48 – 3.33 | <0.001* |
| **Total cholesterol ≥240 mg/dL** | 0.52 | 0.19 – 1.44 | 0.209 |

* Statistically significant result.

**Cox regression – females (observations: 850; deaths: 110)**

| **Neck circumference** | **Hazard ratio** | **95% confidence interval** | ***p* value** |
| --- | --- | --- | --- |
| **Quartile 1 (25 – 34 cm)** | Referent category | | |
| **Quartile 2 (35 – 36 cm)** | 1.18 | 0.69 – 2.02 | 0.549 |
| **Quartile 3 (37 – 39 cm)** | 1.83 | 0.99 – 3.34 | 0.051 |
| **Quartile 4 (40 – 50 cm)** | 4.18 | 1.80 – 9.74 | 0.001* |
| **Age at baseline** | 1.05 | 1.03 – 1.07 | <0.001* |
| **Primary school education** | 1.62 | 0.93 – 2.83 | 0.87 |
| **Body mass index ≥30 kg/m^2^** | 0.93 | 0.88 – 0.98 | 0.012* |
| **Poor physical activity** | 2.43 | 1.50 – 3.94 | <0.001* |
| **Blood pressure ≥140/90 mmHg** | 0.99 | 0.66 – 1.48 | 0.951 |
| **Fasting glucose ≥126 mg/dL** | 1.80 | 1.22 – 2.66 | 0.003* |
| **Total cholesterol ≥240 mg/dL** | 0.63 | 0.33 – 1.18 | 0.145 |

* Statistically significant result.
