# Supplementary material for: Neck circumference as a predictor of all-cause mortality in middle-aged and older adults in rural Ecuador
Source: Int Health. 2024 Jan 17;16(6):664–9. doi: 10.1093/inthealth/ihad119 (PMC11532669; doi:10.1093/inthealth/ihad119)
Supplement: ihad119_Supplemental_Files [file ihad119_supplemental_files.zip › Supplementary file 2.docx]

**Supplementary file 2.** Unadjusted (upper panel) and multivariate (lower panel) Poisson regression models showing no significant association between the waist circumference and mortality risk.

**Unadjusted model**

| **Mortality** | **IRR** | **95% confidence interval** | ***p* value** |
| --- | --- | --- | --- |
| **Waist circumference** | 0.99 | 0.98 – 1.01 | 0.260 |

**Multivariate model**

| **Mortality** | **IRR** | **95% confidence interval** | ***p* value** |
| --- | --- | --- | --- |
| **Waist circumference** | 0.99 | 0.98 – 1.01 | 0.627 |
| **Age at baseline** | 1.06 | 1.04 – 1.07 | <0.001* |
| **Being female** | 0.83 | 0.63 – 1.09 | 0.178 |
| **Primary school education** | 1.13 | 0.78 – 1.64 | 0.515 |
| **Current smoker** | 0.73 | 0.29 – 1.80 | 0.491 |
| **Body mass index ≥30 kg/m^2^** | 0.82 | 0.55 – 1.24 | 0.354 |
| **Poor physical activity** | 2.08 | 1.46 – 2.95 | <0.001* |
| **Poor diet** | 0.95 | 0.56 – 1.62 | 0.851 |
| **Blood pressure ≥140/90 mmHg** | 1.13 | 0.85 – 1.50 | 0.415 |
| **Fasting glucose ≥126 mg/dL** | 1.92 | 1.46 – 2.54 | <0.001* |
| **Total cholesterol ≥240 mg/dL** | 0.58 | 0.34 – 0.98 | 0.040* |

* Statistically significant result.
